# Knowledge and practices of postmenopausal women regarding vitamin D and osteoporosis

**DOI:** 10.61622/rbgo/2026rbgo8

**Published:** 2026-03-20

**Authors:** Yasmin Bastos-Silva, Luiza Borges Aguiar, Luiz Francisco Baccaro, Adriana Orcesi Pedro, Lúcia Costa-Paiva

**Affiliations:** 1 Universidade Estadual de Campinas Faculdade de Ciências Médicas Departmento de Tocoginecologia Campinas SP Brazil Departmento de Tocoginecologia, Faculdade de Ciências Médicas, Universidade Estadual de Campinas, Campinas, SP, Brazil.

**Keywords:** Menopause, Postmenopause, Osteoporosis, Osteoporosis, postmenopausal, Bone density, Calcium, dietary, Knowledge, Vitamin D

## Abstract

**Objective:**

Vitamin D has a direct influence on musculoskeletal health, and vitamin D deficiency is related to lower bone density and greater bone resorption and to a higher incidence of fractures. To assess knowledge, attitudes and practices regarding vitamin D and osteoporosis in postmenopausal women.

**Methods:**

This was a cross-sectional study carried out with 260 postmenopausal women who underwent routine consultations at the menopause outpatient clinic of Campinas State University-São Paulo. Women aged 50 years or older with amenorrhea for at least 12 months were included. From an interview, sociodemographic information and health data were collected, and knowledge about vitamin D, habits related to vitamin D, and knowledge about osteoporosis were evaluated using the Facts on Osteoporosis Quiz (FOOQ) questionnaire.

**Results:**

A total of 67.31% of women answered less than half of the questions about knowledge of vitamin D, 58.85% performed their day-to-day activities mostly outdoors, and 53.85% said they did not expose themselves sufficiently to the sun in their daily lives. The average knowledge score on the FOOQ was 15.83 (± 3.75). The variables education level, number of correct FOOQ answers and total self-care score were associated with the highest score for knowledge of vitamin D.

**Conclusion:**

Knowledge about vitamin D and osteoporosis in this group of postmenopausal women was limited. High levels of education, knowledge about osteoporosis and self-care were factors associated with knowledge about vitamin D.

## Introduction

According to reviews by the International Osteoporosis Foundation (IOF), low levels of vitamin D are prevalent even in sunny countries, and lifestyle changes, mostly including more time indoor environments, have caused insufficient levels of vitamin D, which has become a global problem.^(1)^ The prevalence of hypovitaminosis D is variable on several continents and is higher in some countries in Europe and Asia.^(1)^

The prevalence of inadequate vitamin D levels (25(OH) D <75 nmol/L), identified through an international epidemiological investigation, in postmenopausal women with osteoporosis was 42% in Brazil, compared to 67% in Mexico and 50% in Chile, with values of 25(OH)D being lower in Mexico.^(2)^

A Brazilian study carried out in the region of São Paulo observed insufficient and deficient levels of vitamin D in the elderly population, being even lower in women than in men. The high prevalence of hypovitaminosis D was surprising considering the high solar incidence in the region, being even higher than in countries such as England, New Zealand and Germany.^(3)^ A high prevalence of hypovitaminosis D was found in several regions of the country, mainly in elderly and postmenopausal women, reaching 99% in elderly individuals and 93.65% in postmenopausal women specifically.^(4)^

After 50 years of age, hypoestrogenism leads to changes in bone metabolism, increasing the risk of osteoporosis. The involvement of bone mass predisposes women to a higher risk of fractures and thus leads to functional disability and worsening quality of life. The mortality rate between the first twelve months of hip fracture is 25%.^(5-7)^

Vitamin D has a direct influence on musculoskeletal health, as it is of fundamental importance for the balance of calcium and phosphorus levels in the body;^(8)^ vitamin D deficiency is related to lower bone density and greater bone resorption, as well as a higher incidence of fractures.^(9)^ Vitamin D (1,25-dihydroxyvitamin D3) is important for bone mineralization and is responsible for stimulating, through a passive process, the absorption of calcium and phosphate through the intestine, depending, of course, on the availability of adequate calcium and vitamin D.^(9)^ Adequate levels of vitamin D favour the absorption of approximately 30% of calcium from the diet, increasing to 80% during periods of growth and pregnancy. In people with vitamin D deficiency, no more than 10 to 15% of calcium is ingested.^(10)^

The main source of obtaining vitamin D is endogenous production through skin synthesis from sun exposure, from the precursor 7-dehydrocholesterol, in its D3 form (cholecalciferol).^(10)^ It can also be obtained in much smaller proportions exogenously through foods such as fish oil and egg yolk or in its D2 form (ergocalciferol) from a fungal steroid present in mushrooms. However, the two are metabolized equally to change their active form to 1,25-dihydroxyvitamin D3 [1,25 (OH) 2D3].^(8,10)^

Studies on vitamin D knowledge among young people show that knowledge in general is low and not accompanied by attitudes and practices that ensure adequate levels of vitamin D. ^(11-13)^ Another study showed that even though the population had adequate knowledge about the benefits of sun exposure, the majority of individuals had deficient levels of vitamin D.^(14)^

Research carried out with women with postmenopausal osteoporosis has shown that even those who were being treated for the disease had inadequate levels of vitamin D and low calcium intake, so women should be educated about optimizing their calcium and vitamin D intake, increasing adherence to treatment and thereby improving their response to osteoporosis treatment.^(15)^

Knowledge about the various aspects of osteoporosis is one of the important factors for assuming preventive attitudes towards the disease. In Brazil, little is known about the level of knowledge and attitudes of women regarding consumption and ideal sun exposure for the acquisition of adequate values of vitamin D as well as its importance as a preventive measure. This study aimed to assess the knowledge, attitudes and practices of postmenopausal women regarding vitamin D and osteoporosis.

## Methods

This study is the result of a survey of postmenopausal women in which aspects of self-care management, risk factors for osteoporosis, calcium intake and knowledge about osteoporosis and vitamin D were evaluated. The sample size calculation was performed using the estimated average total score of the revised self-care agency rating scale (ASAS-R), using the standard deviation value obtained with a pilot sample of the first 30 women interviewed; considering the level of alpha significance or type I error to be 5% (alpha = 0.05) (or 95% confidence interval) and a sampling error of 1.0, the calculated sample was n = 254 women.

This cross-sectional study was carried out from April 2018 to March 2020, with 260 postmenopausal women who attended a routine consultation at the Menopause Outpatient Clinic of the Hospital of the Campinas State University-São Paulo. As inclusion criteria, women had to be 50 years old or older, amenorrhea for at least 12 months and followed up in specialty outpatient clinics at the State University of Campinas-SP. Those patients with cognitive difficulties who were unable to answer the questionnaire were excluded.

A structured interview was conducted using a questionnaire containing 100 questions with comprehensive variables, resulting from a survey that sought to assess aspects of self-care management, risk factors for osteoporosis, calcium intake and knowledge about osteoporosis and vitamin D in postmenopausal women.

The dependent variable of this study was the evaluation of knowledge, attitudes and practices regarding vitamin D. These 27 questions were developed from a literature review on vitamin D^(4,10,16,17)^ and information regarding the sources of vitamin D and its health benefits, levels of sun exposure, and factors that influenced its production and supplementation. In total, 11 questions about knowledge and 16 about attitudes and practices regarding vitamin D were developed. To quantify knowledge, 1 point was assigned for each correct answer, and 0 was assigned for errors, totaling a maximum of 11 points.

The independent variables collected included data such as age, body mass index, sociodemographic characteristics, skin color and sensitivity (Fitzpatrick skin type classification),^(18)^ history of chronic diseases, multimorbidities, history of medication use, reproductive characteristics, use of hormone replacement therapy, age at initiation of hormone replacement therapy, duration of hormone replacement therapy, surgery for ovarian or uterus removal, densitometric diagnosis of osteopenia/osteoporosis, recurrent falls, family history of fracture; dietary calcium intake assessed by the International Osteoporosis Foundation's calcium calculator,^(19)^ self-care agency through the ASAS-R (Appraisal of Self-Care Agency Scale-Revised) questionnaire,^(20)^ alcoholism history, smoking history, level of physical activity, use of calcium and vitamin D supplementation, self-perceived health, fracture risk factors and fracture risk calculated by the Fracture Risk Assessment Tool (FRAX-Brazil).^(21)^

Women's knowledge about osteoporosis was assessed using the Facts on Osteoporosis Quiz (FOOQ) instrument, which consists of a questionnaire of 25 questions that address self-care, risk factors and prevention attitudes associated with osteoporosis, with three answer options: true, false or I don't know. The maximum final score to be achieved is 25, and high scores indicate greater knowledge of osteoporosis

The profile of the study sample was calculated by the mean of absolute frequency (n) and percentage (%) of the categorical variables, and the numerical variables are described as the mean, standard deviation, minimum and maximum values, median and quartiles.^(23)^

The comparative analysis of categorical variables was performed using the Chi-square test or Fisher's exact test (for expected values less than 5). To compare numerical variables, the Mann-Whitney test (for 2 categories) was used.^(23,24)^

The number of vitamin D knowledge correct answers was divided from the median into two categories of 0-3 and 4-11. To analyse the factors associated with the highest number of correct answers to vitamin D knowledge questions, univariate and multiple Poisson regression analyses were performed (with stepwise criteria for variable selection). The level of significance adopted for the statistical tests was 5%, which is P <0.05. Statistical analysis was performed using the Statistical Analysis Software (SAS) program.^(25-28)^ All study participants signed an informed consent form approved by the research ethics committee, CAAE number 46857014.5.0000.5404; official opinion number 2.367.062.

## Results

Table 1 presents clinical and sociodemographic characteristics. A total of 260 women, with a mean age of 62.13 years (± 8.05), participated in the survey; 55.38% had completed primary education, the women had been menopausal for an average of 17.49 years (± 9.83), 81.54% did not use HRT, and most declared themselves as non-white (59.23%) and were classified as having type 1 skin type (22.31%). Regarding the antecedents, osteoporosis/osteopenia was the most prevalent diagnosis, present in 79.17% of women.

**Table 1:** Clinical and sociodemographic characteristics of postmenopausal women studied

Variables		n(%)
Age		
	50-59	116(44.62)
	60-69	98(37.69)
	≥70	46(17.69)
Marital status		
	With companion	146(56.15)
	Without companion	114(43.85)
Education		
	Illiterate / Incomplete primary	44(16.92)
	Complete primary / incomplete high school	144(55.38)
	High school /University	72(27.69)
Skin color		
	White	106(40.77)
	Not White	154(59.23)
Skin sensitivity		
	Type 1	58(22.31)
	Type 2	27(10.38)
	Type 3	54(20.77)
	Type 4	25(9.62)
	Type 5	49(18.85)
	Type 6	47(18.08)
Use of hormone replacement therapy		
	User	48(18.46)
	Ex user	109(41.92)
	Never	103(39.62)
Background		
	Systemic arterial hypertension	125(48.26)
	Diabetes mellitus	48(18.85)
	Dyslipidemia	94(36.15)
	Depression	71(27.31)
	Stroke /Angina	32(12.30)
	Thyroid disease	51(19.62)
	Osteoporosis /Osteopenia	190(79.17)
	Osteoarthritis /Arthritis	130(50.0)

The average ASAS-R women's self-care score was 62.27 (± 9.64). Table 2 shows the frequency of correct answers per item in the FOOQ questionnaire. The lowest frequency of correct answers was in item 9 (7.69%), and the highest was in item 17 (95.38%). The average knowledge about osteoporosis assessed by the FOOQ was 15.83 (± 3.75), with the maximum score being 25.

**Table 2:** Percentage of correct answers about knowledge of osteoporosis assessed by the FOOQ (Facts on Osteoporosis Quiz)

FOOQ	n(%)
1. One in four women over the age of 60 will develop osteoporosis.*	224(86.15)
2. Inactivity increases the risk of osteoporosis.*	228(87.69)
3. Heredity does not play a role in osteoporosis.	72(27.69)
4. Early menopause, such as after a hysterectomy, is not a risk factor for osteoporosis.	131(50.38)
5. High caffeine intake (more than 2 cups per day) increases the risk of osteoporosis.*	174(66.92)
6. A lifetime low intake of calcium will increase the risk of osteoporosis.*	231(88.85)
7. Young women need the equivalent in calcium of a glass of milk a day to prevent osteoporosis.	45(17.31)
8. Smoking is not a risk factor for osteoporosis.	183(70.38)
9. Thin women are more often affected by osteoporosis than heavy women.*	20(7.69)
10. Weight-bearing exercise such as walking can help prevent osteoporosis.*	206(79.23)
11. After age 40, it is too late for people to increase their calcium intake to prevent osteoporosis.	182(70)
12. There is no treatment for osteoporosis once you develop it.	160(61.54)
13. After menopause, osteoporosis may be slowed down bytaking estrogen.*	135(51.92)
14. All individuals lose bone mass after 40 years of age.*	205(78.85)
15. African Americans have a higher risk of osteoporosis than Asian-Americans.	130(50)
16. Normally, bone loss slows down after menopause.	126(48.46)
17. A diet high in calcium throughout life can help prevent osteoporosis.*	248(95.38)
18. Women over 40 need about 1500 mg of calcium.*	140(53.85)
19. There is no way to prevent osteoporosis.	199(76.54)
20. White women are less affected by osteoporosis than African American women.	169(65)
21. Dairy products are a major source of calcium.*	217(83.46)
22. It is normal for bone loss to continue throughout life.*	194(74.62)
23. Active women are at higher risk for osteoporosis than inactive women.	216(83.08)
24. Alcohol abuse is not linked to the incidence of osteoporosis.	165(63.46)
25. A risk factor for osteoporosis is having a mother with it.*	116(44.62)
FOOQ score (mean/ SD)	15.83(±3.75)

* True answers

Table 3 presents women's knowledge about vitamin D. One hundred seventy-five women (67.31%) answered 3 or fewer questions correctly, out of a total of 11 questions.

**Table 3:** Evaluation of the vitamin D knowledge of the women in this study

Variables		n(%)
Already heard about vitamin D		251(96.91)
Where did you hear about vitamin D		
	Professionals	169(67.33)
	Media	37(14.74)
	Professionals and media	21(8.37)
	Friends/Relatives	16(6.37)
	Others	6(2.39)
Benefit of Vitamin D		
	Bone health *	148(58.96)
	Rickets prevention *	4(1.59)
	Prevention of generalized weakness *	24(9.56)
	Chronic disease prevention *	11(4.38)
Sources of Vitamin D		
	Sun *	112(44.62)
	Foods	50(19.92)
	Don't know any source	121(46.72)
Know food sources of vitamin D *		17(6.77)
Season of the year that facilitates the production of vitamin D		
	Spring/ Summer *	73(29.08)
	Fall/ Winter/ Don't know	38(15.14)
	Recognize that sunlight can produce vitamin D *	112(44.62)
Sun exposure time to obtain vitamin D		
	10-20 *	33(13.15)
	>20/ Don't know	79(31.47)
	Did not answer	139(55.37)
Skin color can influence vitamin D production		
	Yes *	35(13.94)
	No/ Don't know	77(30.68)
Know which skin produces vitamin D more easily		
	Clear *	8(3.19)
	Dark / Don't know	27(10.76)
Vitamin D knowledge score		
	0-3	175(67.31)
	4-11	85(32.69)
	Vitamin D knowledge score (mean±SD)	2.42(± 2.22)

* Knowledge questions about vitamin D that were scored.

Table 4 presents attitudes and practices of the women in the study regarding vitamin D. Regarding habits that facilitate production of vitamin D, 58.85% of women performed their daily activities in closed environments, and the majority confirmed that they did not expose themselves sufficiently to the sun (53.85%), mainly because of a lack of time (16.15%). When exposed to the sun, the most exposed parts of the body were the arms (77.69%) and the face (68.08). The majority (68.46%) of the women reported using sunscreen, the majority with daily frequency (57.63) and 55.77% used some measure to protect themselves from the sun, such as a hat (30%), sunglasses (25.38%), parasol (25.38%) or protective clothing (2.31%). The average number of minutes per week that they were exposed to the sun between 10 am and 4 pm from Monday to Friday was 70.75 ± 88.90; on the weekend, this value increased to 73.73 ± 95.46. Most of the women in this study (67.18%) were users of vitamin D supplements.

**Table 4:** Attitudes and practices of the women in the study regarding vitamin D (n = 260)

Variables		n(%)
Environment that carries out the activities		
	Closed	153(58.85)
	Open air	107(41.15)
Woman's opinion about her exposing herself sufficiently to sunlight		
	Yes	120(46.15)
	No	140(53.85)
Reason for not exposing yourself to sunlight		
	Out of time	68(26.15)
	Do not like	43(16.54)
	Its make bad	12(4.62)
	Others	17(6.54)
Most exposed body part when it gets sun		
	Face	177(68.08)
	Hands	16(6.15)
	Whole arms	202(77.69)
	Whole legs	91(35.00)
	Other parts or whole trunk	12(4.62)
Use of sunscreen		178(68.46)
Frequency of using sunscreen		
	Eventual/ Non-daily	75(28.85)
	Daily (7x/ week)	102(57.63)
Use other measures to protect themselves from the sun		145(55.77)
	Hats	78(30.00)
	Sunglasses	66(25.38)
	Umbrella	66(25.38)
	Protective clothing	6(2.31)
Outdoor time between 10 am to 4 pm Monday to Friday (mean ± SD) (minutes / week)		70.75(± 88.90)
Outdoors time between 10 am and 4 pm weekend (mean ± SD) (minutes / week)		73.73(± 95.46)
Use of vitamin D supplementation		
	Yes	174(67.18)
	No	78(30.12)
	Don't know	7(2.70)
Use of calcium supplementation		198(76.45)
	Yes	198(76.45)
	No	61(23.55)
Daily calcium intake (mean ± SD)		391.28 (±265.94)

Table 5 presents the factors associated with greater knowledge of vitamin D. The variables significantly associated with greater knowledge of vitamin D, obtained through multiple regression analysis, were a higher level of education, number of correct FOOQ answers and a higher total self-care score. Participants who had completed high school/university had high scores for knowledge of vitamin D, with PR = 9.12 (95% CI 2.20 - 37.85); women who had the highest number of correct answers on the FOOQ (PR = 1.106; 95% CI 1.029 - 1,187) and the highest total self-care scores (PR = 1.030; 95% CI 1.002 - 1,058) also had the highest vitamin D knowledge score.

**Table 5:** Factors associated with greater knowledge of vitamin D in the Poisson multiple regression analysis

Selected variables	Categories	p-value	P.R. *	95% CI P.R.
Degree of education	Illiteracy/ Incomplete Primary (ref.)	---	1.00	---
	Complete Primary/ Incomplete High School	0.027	4.98	1.20 – 20.63
	High School/ College	0.002	9.12	2.20 – 37.85
FOOQ score	Continuous variable	0.006	1.106	1.029 – 1.187
ASAS-R score	Continuous variable	0.035	1.030	1.002 – 1.058

* PR= Prevalence ratio for the highest score of knowledge of vitamin D (variable categorized by the median as: ‘0-3’ n = 168 or ‘4-11’ n = 82). 95% CI PR = 95% confidence interval for the prevalence ratio. Stepwise criterion for selecting variables. Ref .: reference level

## Discussion

This study evaluated the knowledge of postmenopausal women about vitamin D, in addition to their lifestyle habits related to vitamin D production. Knowledge about vitamin D was insufficient and was associated with schooling, knowledge of osteoporosis and self-care management.

Slightly more than half of the women (58.96%) were able to identify that one of the benefits of vitamin D is bone health. Only 44.62% knew how to identify the sun as a promoter of vitamin D production. Few women knew that vitamin D can be obtained through food (19.92%), and only 6.77% knew what such foods were. Only 13.15% agreed on the recommended daily sun exposure time for adequate vitamin D synthesis. Similar data were found in a survey in Canada in which few research participants were able to identify the food sources of vitamin D and only 14% were able to provide information about the amount of sun exposure time necessary to produce vitamin D.^(11)^

The frequency of sunscreen use was moderate (68.46%), and more than half of participants used it daily. There has been much questioning about the benefits and risks of sun exposure necessary to produce vitamin D versus the risk of skin cancer, especially considering the ideal times for exposure with a high incidence of UVB rays. Kung et al.^(29)^ observed in their study that even for middle-aged women in China who were aware of the benefits of sunlight, knowledge about the harmful effects of the sun restricted their exposure to the sun. According to the recommendation of the Brazilian Society of Dermatology, it is possible to expose yourself to the sun with care, in a light and gradual way, avoiding burns and skin cancer and minimizing ageing, to benefit from the well-being that it provides to us. In addition, direct exposure of covered areas, such as the legs, back, stomach, or palms and soles of the feet, for 5 to 10 minutes every day, was encouraged to synthesize vitamin D without overloading the areas chronically exposed to the sun. The adoption of practices that minimize the intensity of sun exposure reduces the chance of developing skin cancer by 50% in individuals at risk such as those with lighter skin who must take additional protective measures.^(30)^

The level of knowledge of women in this study was insufficient, since approximately two-thirds of the sample answered a very low number of questions (≤0-3 questions). These data corroborate a study carried out in France^(31)^ with the general population aged ≥18 years in which individuals had low levels of vitamin D and 92% of the subjects reported having heard about vitamin D. Women demonstrated better knowledge about the sources of vitamin D as well as the participants with the best educational levels and income. The sun was cited as a source by 72% of the participants, higher than this study. Older participants, women and those already using vitamin D supplementation were able to associate vitamin D with bone health and osteoporosis. The French study observed a strong influence of information sources and social and economic factors on knowledge.^(31)^ Studies from Canada^(11)^ and the Middle East^(12,13)^ also showed poor knowledge about vitamin D in young populations. One of the few studies that assessed knowledge in postmenopausal women also showed that most had heard about vitamin D, but few knew about its sources and benefits.^(29)^

Unlike this study, a survey conducted in the United Kingdom^(32)^ found a good level of knowledge in the general population between 18 and 65 years old, with the majority identifying sources of vitamin D such as the sun, food and supplements. However, as in our research, knowledge about which specific foods are sources of vitamin D was poor.

Educational measures appear to be effective in improving knowledge. Goodman et al.^(33)^ carried out an educational intervention on vitamin D through an application in a population of young adults in Canada and observed that there was a more marked increase in the knowledge of vitamin D in more educated individuals after receiving guidance through an app. However, it remains to be seen whether such apps are sufficient to change attitudes and practices.

Knowledge about the disease can assist in adherence to treatment and health promotion. In the present study that applied the FOOQ, the mean score was 15.83 out of a total possible score of 25, indicating more than 50% correct answers for most questions. Knowledge about osteoporosis varies in different populations; in the United States, 90% of American women have heard about the disease.^(34)^ A study carried out in the Brazilian population showed that the assessed knowledge obtained through the OPQ (Osteoporosis Questionnaire) was 3.78 for a maximum score of 20 points, thus demonstrating limited knowledge.^(35)^

In this study, regression analysis showed that greater knowledge about osteoporosis was associated with greater knowledge of vitamin D although it showed a small association (PR = 1.106, 95% CI 1.029 – 1.187). This finding can be attributed to the fact that this study sample is from a population of postmenopausal women who are followed up in a specialized outpatient clinic focused on menopause and osteoporosis. The association between the two types of knowledge has not been evaluated in the literature. However, it is believed that postmenopausal women should be guided and have knowledge about the most prevalent diseases at this stage of life.

Greater self-care management was also shown to be related to greater knowledge of vitamin D, assuming that those who have greater knowledge about important factors related to the most prevalent diseases in this stage of life tend to have a greater ability to meet and to recognize their own needs and act appropriately to promote and to manage their health.

Furthermore, it is essential to highlight the significant variability in solar exposure depending on the tropical latitudes in different regions. In summer, the sun's rays reach the earth from a higher position, while in winter, the sun appears lower and less radiation is scattered. In contrast, the sun appears low in the sky during winter, spreading its rays over a much wider area, making it less effective. Since Brazil is a large country, there are large seasonal variations in exposure to solar rays and vitamin D in regions from the north to the south, in addition to aspects related to dietary habits and skin pigmentation. These differences may also be present in the importance and knowledge about vitamin D.

The present study has some limitations. The first aspect is the fact that it is a population of women who attend a specialized clinic within a hospital service and may not represent the general population. Another fact is that it is a cross-sectional study, so the causal relationship between the associated factors and the knowledge of vitamin D cannot be clearly established. Prospective studies or clinical trials would be important to better establish a cause-and-effect relationship. In addition, it is important to highlight that we did not use a validated questionnaire about knowledge, attitudes and practices about vitamin D, as there is no validated questionnaire in our population. Using validated questionnaires in the local language is important to ensure that participants correctly understand the questions and that the answers accurately reflect their opinions and experiences. They also allow for greater precision and reliability in what is intended to be measured, minimizing errors and biases in the answers and allowing for greater comparability with other scientific studies. Despite this, we emphasize the importance of assessing the knowledge and attitudes of postmenopausal women about vitamin D, considering the culture, season, latitude and particularities of each country.

## Conclusion

This study showed that knowledge about vitamin D and osteoporosis in postmenopausal women was insufficient. Greater knowledge about vitamin D was associated with a higher educational level, knowledge about osteoporosis and greater self-care. Further research is needed to investigate knowledge of and attitudes about vitamin D, including a sample with a representative population, so that educational measures and public health strategies can be implemented to maintain the bone health of postmenopausal women. Structured questionnaires need to be developed, validated and standardized to enable reproducibility in different populations.

## Data Availability

The research data are described in the article presented.
